# Can the type of gallstones be predicted with known possible risk factors?: a comparison between mixed cholesterol and black pigment stones

**DOI:** 10.1186/1471-230X-14-88

**Published:** 2014-05-06

**Authors:** Harshi TW Weerakoon, Jamburagoda GS Ranasinghe, Ayanthi Navaratna, Ramaiah Sivakanesan, Kuda B Galketiya, Shanthini Rosairo

**Affiliations:** 1 Department of Biochemistry, Faculty of Medicine and Allied Sciences, Rajarata University of Sri Lanka, Saliyapura, Sri Lanka; 2 Department of Biochemistry, Faculty of Medicine, University of Peradeniya, Peradeniya, Sri Lanka; 3 Department of Chemistry, Faculty of Science, University of Peradeniya, Peradeniya, Sri Lanka; 4 Department of Surgery, Faculty of Medicine, University of Peradeniya, Peradeniya, Sri Lanka

**Keywords:** Gallstones, Risk factors, Mixed cholesterol, Black pigment

## Abstract

**Background:**

Pathogenesis of gallstones (GS) is multifactorial and multiple genetic and environmental factors have been identified in different populations for different types of GS with varying prevalence. However the role of the each aetiological factor on the formation of mixed cholesterol and black pigment GS has not being addressed adequately. Hence in this study we attempted to compare known possible risk factors for mixed cholesterol and black pigment GS among two groups of patients with two types of GS.

**Methods:**

The study was done on a cohort of patients with symptomatic GS admitted to the Teaching Hospital Peradeniya, Sri Lanka over a period of 18 months. Clinical and epidemiological data and physical parameters of the patients were recorded and surgically removed GS were analyzed chemically and physically to identify the type of GS. In addition lipid profile was done in all the patients with normal serum bilirubin levels.

**Results:**

A total of 86 patients were included in the study. Mixed cholesterol GS was significantly common among females than males (χ^2^ test, p = 0.029). Mixed cholesterol GS was commonly seen among patients belonging to Moor ethnicity (χ^2^ test, p = 0.009). Majority of patients with mixed cholesterol GS had body mass index above 25 kg/m^2^ (χ^2^ test, p = 0.018). Black pigment GS were significantly common among patients with type II diabetes mellitus (Fisher’s exact test, p = 0.035). Further all the patients with chronic haemolytic anaemia and alcoholic cirrhosis had black pigment GS. Age, family history, Fasting Blood Glucose, dyslipidaemia, lipid profile, parity and use of oral contraceptive pills in females, smoking and alcohol intake in males did not differ significantly among patients in the two groups.

**Conclusion:**

Gender, ethnicity and body mass index can be used to predict the formation of mixed cholesterol GS and black pigment GS.

## Background

Gallstone (GS) disease is one of the leading upper gastrointestinal surgical problems in the Western countries causing a significant health care burden [1]. Though prevalence of GS disease is low among Asians a rising trend of GS disease is observed [2]. The worldwide distribution of types of GS is different and cholesterol GS is the commonest type in Western population [3] while brown pigment GS is the commonest type in East Asians [4]. Black pigment stones are rare in these countries. Compare to Western and East Asian countries black pigment GS are prevalent in South Asian population [3]. Moreover, of the different types of cholesterol GS, mixed cholesterol and black pigment GS are identified as the predominant types of GS in South Asian population [5,6]. Therefore the identification of aetiopathogenesis of GS with special reference to both mixed cholesterol and black pigment GS is important in implementing preventive measures for GS among South Asians. Both mixed cholesterol and black pigment stones are composed of same chemical constituents namely cholesterol, calcium bilirubinate, calcium phosphate and calcium carbonate [7]. Cholesterol is the main chemical constituent in mixed cholesterol GS whereas calcium bilirubinate is the main constituent in black pigment GS [7]. Further in mixed cholesterol GS, cholesterol is concentrated mainly in peripheral zones and pigments are mainly seen in the core [7]. Therefore though these two types of GS are described separately existence of common aetiological factors for both types of GS is also a possibility.

Main cause for the pathogenesis of black pigment GS is the elevated unconjugated bilirubin level in bile [8]. Conditions causing chronic hemolysis and chronic liver disease are identified as classic causes for elevated biliary unconjugated bilirubin [9]. Apart from that gall bladder hypomotility [10], acquired enterohepatic circulation of unconjugated bilirubin [8] and effects of trace elements [11] are under investigation as the causes for pathogenesis of black pigment GS, as most of the incidence of this type of stone cannot be explained by the classic causes. Of these causes the acquired enterohepatic circulation of unconjugated bilirubin is a new concept of pathogenesis of black pigment GS and ileal dysfunction is identified as the principle pathology behind this concept [8]. Diseases, resection or bypass surgeries of ileum, dietary factors causing impaired ileal function (high carbohydrate and cholesterol) and alcoholism are the main causes for ileal dysfunction [8].

Pathology of cholesterol GS is primarily associated with biliary cholesterol hypersecretion [12]. Apart from that bile salt concentration and composition, biliary phospholipid concentration, nuclear promoting and inhibiting proteins and gall bladder and intestinal motility are identified as the other causes of cholesterol supersaturation and crystallization [13]. Genetic predisposition, female gender, ageing, obesity, metabolic syndrome, rapid weight loss, high parity, exogenous intake of oestrogen and high calorie diet are identified risk factors for cholesterol GS disease [3]. Effect of smoking and alcohol consumption on pathogenesis of cholesterol GS is also being evaluated at present.

Though there are such well identified aetiological factors for the pathogenesis of cholesterol and black pigment GS, the aetiopathogenesis of mixed cholesterol and black pigment GS is still unclear among South Asians. Studies done outside South Asia have revealed that most of the known risk factors cannot be used to differentiate the chemical composition of GS clinically [14,15]. As mixed cholesterol and black pigment GS are highly prevalent among South Asians, the identification of predicting factors based on a South Asian population is important when describing the aetiopathogenesis of these two types of GS. In the present study we sought to compare the effect of known possible aetiological factors on the formation of mixed cholesterol and black pigment GS in a cohort of patients with the two types of GS.

## Methods

The study was carried out among adult (age > 18 years) symptomatic patients with GS admitted to Teaching Hospital, Peradeniya, Sri Lanka for surgical removal of GS over a period of 18 months. A total of 102 patients underwent GS removal surgeries during the study period and the relevant data of each patient were recorded in an examiner administered individual formatted data sheets after obtaining informed written consent. These include demographic data (age, gender and ethnicity) and the presence of known aetiological factors for GS disease. The considered aetiological factors include age, gender, smoking, alcohol consumption, family history of GS disease and comorbid conditions (type II diabetes mellitus - DM, dyslipidaemia, chronic haemolytic anaemia, chronic liver disease, ileal resection and ileal bypass surgeries). Females were additionally questioned on parity and use of exogenous oesterogen therapy (oral contraceptive pills or hormone replacement therapy). Number of still and live births were considered to determine the parity.

Weight and height of the patients were measured using standard techniques and body mass index (BMI) was calculated. According to the BMI values, patients were classified into two groups as normal, overweight or obese. The categorization was done using WHO BMI cutoff values given for the South Asian community and whole world. According to the cutoff values for whole world, patients with BMI less than 25 kg/m^2^ were classified into normal and 25 kg/m^2^ or above as overweight and obese [16]. According to the South Asian cutoff values patients with BMI less than 23 kg/m^2^ were classified as normal and 23 kg/m^2^ or above as overweight and obese [17].

Patients with normal serum bilirubin (total) [18] were further tested for serum triglycerides (TG), total cholesterol (TC), HDL-cholesterol and LDL - cholesterol using 2 ml of venous blood obtained after 12 hours of fasting period [19]. Serum lipid parameters were analyzed by enzymatic colorimetric method according to the procedure given in commercially available enzymatic kits purchased from Aggape diagnostics, Germany.

GS were collected at the time of surgery were preserved to identify the physical characteristics. Chemical composition was tested using Fourier Transform Infrared Spectroscopy (Shimadzu IR Prestige 21) technique by previously described methods [20,21]. Based on the results of physical and chemical analysis these were categorized as pure cholesterol, mixed cholesterol, black pigment and brown pigment GS [7]. The possible causative factors were then compared between mixed cholesterol and black pigment GS.

Ethical clearance for the study was obtained from the Ethical Review Committee Faculty of Medicine, University of Peradeniya, Sri Lanka.

Data were analyzed by minitab 16 software to identify the predicting factors of two types of GS. Continuous data were compared by two sample t test (*n* > 30) or Mann Whitney U test (*n* < 30) while categorical data were analyzed by chi square or Fisher’s exact test.

## Results

A total of 102 patients underwent surgical removal for GS during the study period and of them 48 (47%) had black pigment GS, 38 (37%) had mixed cholesterol GS, 10 (10%) had pure cholesterol GS, and 6 (6%) had brown pigment GS. Majority (84%) of the study sample had either mixed cholesterol or black pigment GS.

Majority of this cohort were females (*n* = 66, 77%) and female to male ratio was 3:1. Mean ages of females and males with GS disease were 44 ± 10.6 and 52 ± 11.3 years respectively with a statistically significant difference (Two sample t test, p = 0.008). Age range of the study group was 27 – 82 years with mean age of 45.95 (±11.28) years.

Of the 66 females, 33 patients (50%) had mixed cholesterol GS while out of 20 males only 05 (25%) had mixed cholesterol GS. Compared to males, development of mixed cholesterol GS was significantly common among females (χ^2^ test, df = 1, p = 0.029). Mean ages and the gender distributions between these two groups were not significantly different (Table 1).

**Table 1:** Comparison of the age and gender between mixed cholesterol GS and black pigment GS

**Description**	**Age at presentation (mean ± SD) years**		
	**Mixed cholesterol GS**	**Black pigment GS**	**P value**
Total group	43.58 ± 11.80	45.00 ± 12.65	0.087*
Females	41.97 ± 09.81	46.10 ± 11.20	0.117*
Males	54.20 ± 18.86	51.25 ± 08.23	0.934^ψ^

*Two sample t test.

^ψ^Mann Whitney U test.

Majority of the group were Sinhalese (*n* = 67, 78%) with twelve Moors and seven Tamils. Compared to Sinhalese patients, occurrence of mixed cholesterol GS was more common among Moors (χ^2^ test, p = 0.009).

Comparison of known aetiological factors for the two types of GS is summarized in Table 2. Of the 66 females, 21 (32%) were on oral contraceptive pills (OCP). None of the patients were on postmenopausal hormone replacement therapy. Frequency of using OCP was not significantly different among females in two groups (p = 0.173). Statistically significant differences was not observed (p = 0.649) for parity as well. Only 6 (7%) patients had a positive family history and was not significantly related to the two type of GS (p = 0.083).

**Table 2:** Comparison of aetiological factors between mixed cholesterol GS and black pigment GS groups

**Aetiological factor**	**Mixed cholesterol group (*n*** **= 38)**	**Black pigment group (*n*** **= 48)**	**P value**
Family history of GS disease (*n*, %)			
Positive	05 (13)	01 (02)	0.083^γ^
Negative	33 (87)	47 (98)	
Body Mass Index (*n*, %)			
<25 kg/m^2^	12 (32)	27 (56)	0.018^ψ^
>25 kg/m^2^	26 (68)	21 (44)	
Body Mass Index (*n*, %)			
<23 kg/m^2^	07 (18)	14 (29)	0.224^ψ^
>23 kg/m^2^	32 (82)	34 (71)	
Exogenous oestrogen in females (*n*, %)	12 (36)	09 (27)	0.173^ψ^
Positive			
Negative	21 (64)	24 (83)	
Parity (Mean, SD)	2.97 (1.59)	2.79 (1.58)	0.649*
Smoking in males (*n*, %)			
Positive	04 (80)	08 (53)	0.603^γ^
Negative	01 (20)	07 (47)	
Alcohol consumption in males (*n*, %)			1.000^γ^
Positive	04 (80)	12 (80)	
Negative	01 (20)	03 (20)	
Dyslipidaemia (*n*, %)			0.651^γ^
Positive	03 (08)	02 (04)	
Negative	35 (92)	46 (96)	
Serum total cholesterol (mg/dl)	169.67 ± 25.90	171.47 ± 27.17	0.790*
Serum triglycerides (mg/dl)	153.74 ± 19.02	145.20 ± 26.74	0.153*
Serum HDL-cholesterol (mg/dl)	54.11 ± 9.36	51.18 ± 8.89	0.211*
Serum LDL-cholesterol (mg/dl)	85.96 ± 33.55	85.84 ± 26.62	0.988*
Fasting Blood Glucose (mg/dl)	98.80 ± 19.26	98.01 ± 15.71	0.854*
Type II Diabetes Mellitus (*n*, %)			0.035^γ^
Positive	04 (11)	15 (31)	
Negative	34 (89)	33 (69)	
Chronic haemolytic anaemia			-
Positive	00 (00)	01 (02)	
Negative	38 (100)	47 (98)	
Chronic liver disease			-
Positive	00 (00)	04 (08)	
Negative	38 (100)	47 (92)	

^γ^Fisher’s exact test.

^ψ^Chi Square test.

*Two sample *t* test.

Majority of patients with mixed cholesterol GS (*n* = 26, 68%) had BMI above 25 kg/m^2^ than that of black pigment GS (*n* = 21, 44%) with a statistical significance (p = 0.018). However according to the Asian cutoff values only 7 (18%) patients with mixed cholesterol GS and 14 (29%) patients with black pigment GS had BMI below 23 kg/m^2^ while majority of patients (*n* = 65, 77%) with both mixed cholesterol and black pigment GS had BMI above 23 kg/m^2^.

Further, though majority of females and males with mixed cholesterol GS had BMI above 25 kg/m^2^ compared to BMI of patients with black pigment GS, the difference was not statistically significant (Table 3).

**Table 3:** Comparison of BMI of female and male patients with mixed cholesterol and black pigment GS

**BMI category (kg/m**^**2**^**)**	**Females**		**P value**^**ψ**^	**Males**		**P value**^**γ**^
	**Mixed cholesterol GS *n *** **(%)**	**Black pigment GS *N *** **(%)**		**Cholesterol GS *n *** **(%)**	**Pigment GS *n *** **(%)**	
< 25	10 (30)	17 (52)	0.080	02 (40)	10 (67)	0.347
> 25	23 (70)	16 (48)		03 (60)	05 (33)	

^ψ^χ^2^ test, df-1.

^γ^Fisher’s exact test.

Majority of males in the study group had habits of smoking (*n* = 12, 57%) and alcohol consumption (*n* = 16, 76%) and all were then, present smokers and alcohol consumers. Among the male patients with mixed cholesterol GS, 80% (*n* = 4) had the habit of smoking and alcohol consumption while 53% (*n* = 8) and 75% (*n* = 12) of male patients with black pigment GS had these two habits respectively. However statistically significant differences were not observed for both habits (Table 2).

Type II DM was more common among patients with black pigment GS and it was significantly (p = 0.035) higher compared to patients with cholesterol stones. However, in patients who were not diagnosed to have DM (*n* = 67) the difference in the mean fasting blood glucose (FBG) concentrations between the two groups was not significant (p = 0.854). Only 5 patients in the study group had dyslipidaemia and frequency of dyslipidaemia among patients with two types of GS was not significantly different (p = 0.653). Similar observations were seen for the serum TC, TG, HDL- cholesterol and LDL- cholesterol (*n* = 62).

Only one patient had a history of chronic haemolytic anaemia, while 4 patients had chronic liver disease. Alcohol was the identified cause for chronic liver disease in all 4 patients. All the GS recovered from these patients were black pigment GS. None of the patients in the study groups had history of ileal resection or ileal bypass surgery.

## Discussion

Pathogenesis of GS is multifactorial and depends on the interplay of multiple environmental and genetic risk factors. Though there are well identified aetiological factors for the pathogenesis of cholesterol and black pigment GS, most of the aetiological factors tested in this study cannot be used to predict the type of GS as mixed cholesterol and black pigment GS. This study highlights the possibility of multifactorial effect of known risk factors for the pathogenesis of mixed cholesterol GS and black pigment GS. Female gender, Moor ethnicity and overweight or obesity are identified as the predicting factors of mixed cholesterol GS over black pigment GS. Further to that this study shows a significantly high incidence of black pigment GS among males. Patients with type II DM were also more likely to have black pigment GS.

Although the chemical composition of GS in this community is different to the Western population, occurrence of GS disease is common among females in this community and it is similar to the west [3]. Mean age of development of GS disease in females was around 44 years which was significantly earlier than that of males. Moreover majority of female patients developed the disease before the age of menopause which is usually seen around the age of 50 years [22]. All these facts together highlight the effect of female gender on the pathogenesis of both types of GS. Oestrogen increases the biliary cholesterol secretion causing cholesterol supersaturation [23], while progesterone inhibits the GB contraction and function of sphincter of oddi causing bile stasis [24]. Therefore the effect of progesterone can be considered as the reason to have high incidence of both types of GS among females as pathogenesis of both cholesterol and black pigment GS is associated with gall bladder hypomotility [10]. In the current study cohort, mixed cholesterol GS are significantly common among Moors. The reason for the high prevalence of GS only in some ethnic groups is being evaluating and the current finding might be due to the involvement of either genetic factors, life style factors or both. However further investigations are required to identify the exact reason.

One third of females in the study group were on exogenous oestrogen therapy (OCP) and it did not have significant association with mixed cholesterol or black pigment GS. Exogenous intake of oestrogen is known to increase the risk of developing cholesterol GS [25,26]. Though it is considered as a risk factor, such effect has not been clearly evident in some other recent studies too [27]. Presence of low dose of estrogen in novel pills might be the reason for this observation. Further an effect of parity on the chemical composition of GS was not detected in this study. Parity is identified as a risk factor to develop GS in populations with high incidence of cholesterol GS [28,29]. Similar observations have been discussed in study done in Germany [15]. Pregnancy is a known cause of gall bladder hypomotility [30] and elevated oestrogen level in pregnancy increases biliary cholesterol concentration resulting in sludge formation, the precursor of cholesterol GS. However, it is a reversible process in most of the pregnant women [30]. It might be the reason for the current finding. Parity is not identified as a risk factor among Hispanic population with significantly high prevalence of GS as well [31].

In our study group majority of patients with mixed cholesterol GS had BMI of above 25 kg/m^2^ indicating both overweight and obesity as favorable factors for formation of mixed cholesterol GS. Further, though it was not detected in this study, a study done in Germany [15] identified a significant effect of obesity (BMI > 30 kg/m^2^) on formation of cholesterol GS separately for females and males also. Failure of such identification might be due to the inclusion of lesser numbers of subjects into two gender categories. A strong association of obesity with the development of GS is identified in many case control studies conducted in western population [15,28,32]. Several mechanisms are suggested for high incidence of pure cholesterol GS among obese people. Of them hyperinsulinaemia caused by insulin resistance [33], increased cholesterol saturation index in bile due to the stimulatory effect of insulin on HMG Co A reductase (3-hydroxy-3-methyl-glutaryl-CoA reductase) enzyme [34] are discussed widely. The current study highlights the significant effect of overweight and obesity even for the formation of mixed cholesterol GS. Interestingly when BMI cutoff values for South Asians was considered, majority of patients with both mixed cholesterol and black pigment GS had BMI above 23 kg/m^2^. This indicates a high risk of cardiovascular disease among majority of patients with GS irrespective of the type. Since high morbidity and mortality from cardiovascular diseases has been observed in patients with GS in USA [35] this could be taken as a warning sign for development of cardiovascular disease in future for patients in this cohort as most of the patients were around 40 years.

Positive family history was identified only in 6 (7%) patients of the study cohort and it is a lesser incidence compared to existing literature [3,36]. However in this study positive family history of GS was recorded based on patients awareness alone and hence this results might not be the true representation, as diagnosis of GS is mainly done following development of symptoms where only 1/3rd of people with GS develop symptoms related to GS during their lifetime [3].

Similar to most of the other studies, factors which have direct influence on increased unconjugated bilirubin in bile were found in only few patients with black pigment stones. Majority of patients had obscured reasons to develop black pigment GS. However male gender and type II DM were identified as predicting factors of black pigment GS.

In this study black pigment GS was significantly high in patients with type II DM than mixed cholesterol GS. However, FBG was not significantly different among patients with two types of GS. Therefore the small sample size can be considered as one possibility for the statistically significant difference. Further, DM causes pathogenesis of GS in three ways. Type II DM which is associated with hyperinsulinaemia impairs both gall bladder [37] and intestinal motility [38]. Though DM has an effect on pathogenesis of GS [37-39], previous studies are also not identified it as a strong risk factor. DM is identified as a risk factor in the presence of other risk factors like obesity and family history of GS disease [40]. Moreover in Western population obesity is the strongest risk factor for GS even in patients with DM [41].

Majority of males were then, current smokers and smoking was not associated with the type of GS. However GS had been identified as a disease of nonsmokers in a previous study [42]. Protective effects of cigarette smoke on the pathogenesis of GS [42,43] and absence of such relationship [44,45] also identified in some previous studies indicating controversies of effect of smoking on GS formation. As the exact mechanisms by which the smoking affect the pathogenesis of GS have not been identified, further studies are required to identify the effect of smoking on GS pathogenesis. Similar observations can be seen on the effect of alcohol. It is an area which is explored widely and a positive association between alcoholism and development of pigment GS has been identified in patients without a history of cirrhosis as well [46]. Further a protective effect of alcohol on development of GS is also identified [44,47]. However a significant association between alcohol and the chemical composition of GS was not identified in this study. This fact has been proven in previous studies as well [14].

Abnormalities in lipid homeostasis is identified in patients with cholesterol GS in some studies and it is identified as a risk factor for development of cholesterol GS [31,48]. Interestingly mean values of all the tested lipid parameters were seen in normal range in two groups and none of the tested lipid parameters were identified as predicting factors of pathogenesis of two types of GS. Though abnormalities in serum lipid is identified in patients with cholesterol GS, none of the lipid parameters were related to the occurrence of mixed cholesterol and black pigment GS.

## Conclusion

Mixed cholesterol GS were more common among females, Moors and patients with BMI above 25 kg/m^2^ whereas pigment stones were more common among males and the association of type II DM with the two types of GS needs further exploration . Therefore gender, ethnicity and BMI can be used as the factors to predict the formation of mixed cholesterol GS and black pigment GS.

## Abbreviations

GS: Gallstones; BMI: Body mass index; DM: Diabetes mellitus; OCP: Oral contraceptive pills; FBG: Fasting blood glucose; TC: Total cholesterol; TG: Triglyceride.

## Competing interests

The authors declare that they have no competing of interests.

## Authors’ contributions

HTWW, JGSR, RS and KBG conceived and designed the study. KBG and SR did selection and recruitment of the patients. HTWW did data collection and analysis of serum lipid profiles. HTWW and AN analyzed the chemical composition of gallstones. HTWW, JGSR and RS did data analysis. HTWW drafted the manuscript. All authors read and approved the final manuscript.

## Pre-publication history

The pre-publication history for this paper can be accessed here:

http://www.biomedcentral.com/1471-230X/14/88/prepub
